# Molecular Modelling in Bioactive Peptide Discovery and Characterisation

**DOI:** 10.3390/biom15040524

**Published:** 2025-04-03

**Authors:** Clement Agoni, Raúl Fernández-Díaz, Patrick Brendan Timmons, Alessandro Adelfio, Hansel Gómez, Denis C. Shields

**Affiliations:** 1 School of Medicine, University College Dublin, D04 C1P1 Dublin, Ireland; raul.fernandezdiaz@ucdconnect.ie; 2 Conway Institute of Biomolecular and Biomedical Science, University College Dublin, D04 C1P Dublin, Ireland; 3 Discipline of Pharmaceutical Sciences, School of Health Sciences, University of KwaZulu-Natal, Durban 4000, South Africa; 4 IBM Research, D15 HN66 Dublin, Ireland; 5 Nuritas Ltd., Joshua Dawson House, D02 RY95 Dublin, Ireland; timmons.patrick@nuritas.com (P.B.T.); adelfio.alessandro@nuritas.com (A.A.); gomez.hansel@nuritas.com (H.G.)

**Keywords:** molecular modelling, bioactive peptides, homology modelling, virtual screening, molecular docking, molecular dynamics simulation, AlphaFold, Protein Language Models

## Abstract

Molecular modelling is a vital tool in the discovery and characterisation of bioactive peptides, providing insights into their structural properties and interactions with biological targets. Many models predicting bioactive peptide function or structure rely on their intrinsic properties, including the influence of amino acid composition, sequence, and chain length, which impact stability, folding, aggregation, and target interaction. Homology modelling predicts peptide structures based on known templates. Peptide–protein interactions can be explored using molecular docking techniques, but there are challenges related to the inherent flexibility of peptides, which can be addressed by more computationally intensive approaches that consider their movement over time, called molecular dynamics (MD). Virtual screening of many peptides, usually against a single target, enables rapid identification of potential bioactive peptides from large libraries, typically using docking approaches. The integration of artificial intelligence (AI) has transformed peptide discovery by leveraging large amounts of data. AlphaFold is a general protein structure prediction tool based on deep learning that has greatly improved the predictions of peptide conformations and interactions, in addition to providing estimates of model accuracy at each residue which greatly guide interpretation. Peptide function and structure prediction are being further enhanced using Protein Language Models (PLMs), which are large deep-learning-derived statistical models that learn computer representations useful to identify fundamental patterns of proteins. Recent methodological developments are discussed in the context of canonical peptides, as well as those with modifications and cyclisations. In designing potential peptide therapeutics, the main outstanding challenge for these methods is the incorporation of diverse non-canonical amino acids and cyclisations.

## 1. Introduction

Molecular modelling techniques are a group of computational approaches that can be used to understand the complex relationships between the molecular structure and function of small molecules and biological macromolecules [1,2]. These techniques have the potential to accelerate the design and discovery of new drugs with enhanced therapeutic properties by providing valuable insights into the structural characteristics, dynamics, and interactions at a molecular level [3,4,5,6]. In this review, we provide an introduction to the compositional, structural, and conformational aspects of peptides and of their protein targets and then go on to identify the main molecular modelling methods used in the course of bioactive peptide identification, spanning homology modelling, docking, virtual screening of peptide libraries, molecular dynamics analyses, and machine-learning prediction.

The increase in computational power and the growing availability of vast amounts of structural data have greatly improved molecular modelling techniques. At the same time, improvements in molecular modelling methods have significantly reduced the time and resources needed for hit identification, hit-to-lead optimization, and lead optimisation in drug discovery [7,8]. This progress has contributed to the discovery of a number of FDA-approved drugs, such as Viracept and Zanamivir, both of which were developed using structure-based drug design methods [1,5,7,9,10]. It has also led to the identification of new possible uses for known drugs (repurposing). For example, in silico molecular modelling identified Ouabain, Digitoxin, and Niclosamide as FDA-approved drugs with strong binding patterns to key viral proteins, suggesting they could be effective treatments for COVID-19 [11]. Similarly, through virtual screening and experimental evaluation, cepharanthine, clofazimine, metergoline, imatinib, and efloxate were identified as potential repurposable drugs with the ability to interfere with SARS-CoV-2 viral entry [12].

Bioactive peptides are promising therapeutic alternatives to small molecules due to their biosafety, medicinal properties, and nutritional and cosmetic benefits, as well as their known effectiveness at low concentrations. These peptides are typically short chains, with fewer than 50 amino acids, and exhibit a wide range of biological activities, including antimicrobial, antihypertensive, anti-inflammatory, anticancer, and antioxidant properties [13,14,15,16,17]. Biological systems already use peptides to address various physiological and pharmacological challenges [18,19]. Beyond natural hormone analogues based on peptides, peptide drug candidates are also utilised to interfere with protein–protein interactions and to target or inhibit intracellular molecules [20,21]. Several peptide-based drugs, such as dulaglutide [22], albiglutide [23], liraglutide (Victoza) [24], and glucagon-like peptide-1 (GLP-1) [25], have been approved for clinical use in treating metabolic disorders. Additionally, peptide-based hormone analogues like plecanatide [26], leuprolide (Lupron) [27], goserelin (Zoladex) [28], octreotide [29], and lanreotide [30] are also available for clinical applications. Collectively, these drugs have generated billions of dollars in sales, fuelling the growing interest in bioactive peptides [31].

The traditional discovery and characterisation of bioactive peptides is, however, challenged by their unique inherent structural diversity and dynamic nature, as well as their poor bioavailability [17,19,32,33]. Experimental methods for identifying and characterising bioactive peptides are typically time-consuming and resource-intensive, highlighting the need for more efficient approaches [34,35]. However, these traditional methods can be significantly accelerated by implementing molecular modelling techniques or integrating them with artificial intelligence (AI) solutions. Such methods can predict peptide structures, explore peptide–protein interactions, investigate the structural dynamics of peptides, and repurpose bioactive peptides with known activities [36,37,38,39,40]. Molecular modelling techniques can, therefore, aid in the rational design of peptide-based drugs to improve their activity, facilitate virtual screening of peptide libraries to identify potential drug candidates, and support structure–activity relationship studies to optimize peptides, and possibly also repurpose peptides with known bioactivities [37,41,42,43]. The application of molecular modelling has been particularly noticeable in discovering potential milk-derived bioactive peptides, where techniques like molecular docking and molecular dynamics (MD) simulations have shown great potential in enhancing the efficiency of generating and discovering bioactive peptides [44,45,46].

While molecular modelling methods have been largely successful in the discovery of small-molecule therapeutics, it remains uncertain whether this success can be replicated in the design and discovery of bioactive peptides [37,44,47,48]. Evidence suggests that directly applying small-molecule or protein design strategies is not well-suited for peptide drug design [38,49,50,51,52]. This is primarily due to peptide conformational flexibility, which limits the effectiveness of small-molecule computational methods. Also, the incorporation of nonstandard amino acids and the use of diverse cyclisation chemistries in peptide drug design pose difficulties for traditional protein-modelling software [38]. As a result, customised computational approaches specifically tailored to peptides are needed and are gradually gaining more and more interest.

The recent integration of molecular modelling techniques with artificial intelligence (AI) and machine-learning methods has led to innovative advancements such as AlphaFold’s protein structure prediction and Protein Language Models, which could significantly enhance the bioactive peptide discovery process [44,53,54,55,56,57,58,59,60]. The use of molecular modelling techniques must, however, always be in tandem with rigorous experimental validations to guarantee effectiveness.

## 2. Structural Characteristics of Bioactive Peptides

**Figure 1:**
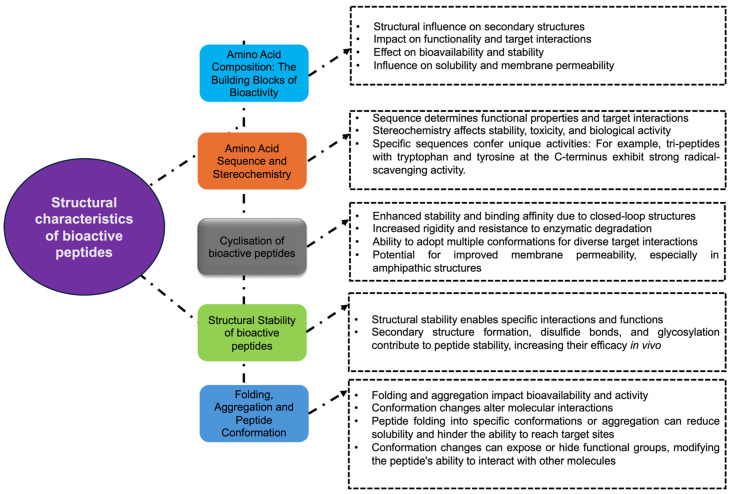
Structural characteristics influencing the bioactivity of peptides. This figure illustrates the key structural features of bioactive peptides and their impact on various functional properties.

The biological activity of any given peptide and its ability to interact with molecular targets are determined by its structural characteristics, such as amino acid composition, sequence, secondary structure, molecular weight, chain length, and the presence of disulfide bonds [16,17,61] (Figure 1). The specific arrangement of amino acids, especially aromatic and charged residues, plays a significant role in determining properties like solubility, binding affinity, and reactivity. These relationships are often elucidated through structure–activity relationship (SAR) studies, which highlight how variations in peptide structure can affect their functional outcomes [62,63,64].

Bioactive peptides typically adopt secondary structures such as α-helices and β-sheets, which can be stabilised through strategies like peptide cyclisation, the introduction of D-amino acids, and covalent cross-linking [16,62,65]. Shorter peptides (2–20 amino acids) with molecular weights between 180 and 1000 Da often exhibit greater bioactivity compared to longer chains, with approximately 42% of the most active peptides having molecular weights below 1000 Da [19,66]. Shorter peptides tend to exhibit higher bioavailability due to their enhanced absorption and reduced susceptibility to proteolytic degradation and overall favourable pharmacokinetic properties [66]. Larger peptides, on the other hand, may exhibit more specific and potent effects compared to smaller peptides or traditional small molecules. This specificity results from the increased number of interactions they can form with target sites, such as through hydrogen bonding and hydrophobic interactions, leading to fewer off-target effects and reduced side effects overall [18,19]. There may be a complex relationship between optimal size and bioactivity, with tumours being modelled to retain larger peptides better and smaller peptides needing to have a higher affinity to be retained in the tumour [67]. Disulfide bonds formed between cysteine residues help stabilise specific secondary structures and maintain the overall conformation of many peptides [62,68,69]. The size of peptides also influences their ability to traverse biological barriers, with smaller peptides generally exhibiting higher permeability, often diffusing passively through lipid bilayers [70,71]. Studies have shown an inverse relationship between peptide size and permeability, with smaller peptides demonstrating higher flux rates across barriers like the Caco-2 cell monolayer [70,72]. As size increases, passive diffusion becomes less effective, and peptides rely more on active transport mechanisms [70]. Medium-sized peptides may utilise specific transporters and/or receptor-mediated transcytosis or function as cell-penetration peptides [70,73]. Larger peptides face significant challenges but can employ strategies such as transcytosis, macropinocytosis, or targeted delivery systems to enhance their transport [74,75]. PepT1 and PepT2, oligopeptide transporters, facilitate the uptake of di- and tripeptides with diverse chemical properties [76]. These oligopeptide transporters also transport neutral tripeptide-like β-lactam antibiotics and other peptide-mimetic drugs [77]. As peptide size increases, the impact of charge on permeability decreases [19,78]. Positively charged peptides generally permeate more efficiently due to favourable electrostatic interactions with negatively charged lipid head groups in membranes. However, as peptide size increases, these interactions become less significant compared to other factors like steric hindrance and hydrophobicity [72,79]. Ongoing research continues to develop innovative strategies to improve the transport of larger peptides, expanding their potential as drug delivery vehicles [80,81].

### 2.1. Amino Acid Composition: The Building Blocks of Bioactivity

Peptide amino acid composition plays a pivotal role in determining its structure and function [82]. Collectively, the composition of amino acids in a peptide influences the peptide’s ability to form secondary structures, such as alpha helices and beta sheets [83,84]. Different amino acids possess varying side chains that can be hydrophobic, hydrophilic, charged, or neutral [83,85,86,87]. The presence of specific amino acids, such as aromatic residues (e.g., tryptophan and phenylalanine) or charged residues (e.g., lysine and glutamic acid), can significantly affect a peptide’s solubility, binding affinity, and reactivity with target molecules [88,89]. Hydrophobic (Val, Ile, and Pro) and positively charged (His, Arg, and Lys) amino acids are enriched with anti-inflammatory peptides [66].

Hydrophobic interactions between amino acids contribute to peptide structure and stability, which can have an important effect on their bioavailability [90,91]. This is particularly important in the gastrointestinal tract, where peptides with a higher proportion of hydrophobic amino acids tend to be more resistant to proteolysis, increasing their bioavailability and allowing them to reach their target sites [92,93,94,95]. On the other hand, polarity determines solubility and the ability of any given peptide to cross biological membranes [96,97,98]. Peptides with a high net charge (positive or negative) tend to interact preferentially with other charged molecules with the opposite sign. The net charge of a peptide, determined by its proportion of positively and negatively charged amino acids, can also influence its interaction with other molecular targets, like extracellular receptors or enzymes [99,100].

In computational analysis, amino acid composition can be quantified to assess how variations in sequence impact biological activity [101,102]. This is particularly important in machine-learning approaches to analyse large datasets of peptide sequences to identify patterns that correlate specific amino acid compositions with desired activities, such as antimicrobial or anti-inflammatory effects [103].

### 2.2. Amino Acid Sequence and Stereochemistry

The order in which amino acids are arranged along a peptide’s backbone plays a significant role in determining its functional properties [16,104,105]. Specific amino acid sequences can confer unique binding affinities and specificities to receptors or enzymes, allowing the peptide to interact with its target molecules and exert its biological effects [106,107,108]. Also, tripeptides with tryptophan and tyrosine at their C-terminus have been shown to exhibit strong radical-scavenging activity, while different combinations of amino acids in tripeptide chains have also shown different antioxidant activities [109,110].

The stereochemistry of a peptide plays a crucial role in determining its stability and resistance to proteolysis. In a study by Lu et al., a modified version of the cationic antimicrobial peptide Pep05 (KRLFKKLLKYLRKF) was examined. In this derivative, all L-lysine and L-arginine residues were substituted with their D-amino acid counterparts. This alteration resulted in significantly enhanced stability and reduced toxicity in vitro. However, the peptide exhibited only minimal activity and caused severe toxicity in vivo [111]. The cruciality of peptide chirality is also established in the report by Chen et al., where the substitution of L-histidine with D-histidine in a peptide reduced the antioxidant activity [112]. Understanding the interplay between amino acid sequence, chain length, and bioactivity is pivotal for the rational design of therapeutic peptides. By elucidating these relationships through molecular modelling, it is possible to design peptides that maintain structural integrity while exhibiting the desired biological activities.

### 2.3. Cyclisation of Bioactive Peptides

The cyclisation of some bioactive peptides, characterised by closed-loop structures, commonly enhances their stability and binding affinity compared to linear peptides [113,114]. Cyclic peptides are typically formed through various cyclisation methods, such as head-to-tail cyclisation, lactamisation, and disulfide bond formation, with the additional structural constraint shown to increase their rigidity and resistance to enzymatic degradation [115,116,117]. Despite this rigidity, many cyclic peptides can adopt multiple conformations in solution, allowing for effective interaction with diverse biological targets [118,119]. Many cyclic peptides are approximately planar, and when they are amphipathic with one hydrophobic face and one hydrophilic or cationic face, this can facilitate interactions with lipid membranes and membrane permeability [120,121,122]. These structural features contribute to the diverse biological activities, including antibacterial and immunosuppressive effects [116,123]. Cyclic peptides are often non-ribosomally synthesised by enzymes encoded in complex bacterial gene clusters, with non-canonical amino acids and linkages that differ extensively from those found in ribosomally encoded peptides [115,124]. mRNA display technology has been harnessed to create screening combinatorial libraries of cyclic peptides that can, in vitro, ribosomally incorporate non-natural amino acids, generating a new space of cyclic peptides to be experimentally screened [125].

### 2.4. Structural Stability of Bioactive Peptides

The ability of a peptide to adopt a stable conformation is essential for its proper interaction with other biomolecules, such as proteins or nucleic acids. A structurally stable peptide is more likely to exhibit predictable and consistent functional properties [126]. It allows for specific binding interactions, enzymatic activity, and efficient targeting of cellular processes [90,127,128]. For example, bioactive peptides intended for food applications or as oral drugs must withstand the harsh acidic and proteolytic conditions of the gastrointestinal tract in order to maintain their structure and exert their biological effects [44,94].

Factors such as secondary structure formation, disulphide bonds, and glycosylation contribute to peptide stability, increasing their efficacy in vivo [129,130,131,132]. The stability of peptides is, therefore, crucial in the pharmaceutical design and development of novel peptides. For example, the more stable GLP-1 peptide analogue semaglutide exhibits a lower degradation rate by DPP4 protease and increased binding affinity to albumin [133]. Several reports have extensively reviewed the available strategies to improve the stability of bioactive peptides [90,91,134]. While molecular modelling is essential for understanding the structural stability of bioactive peptides and offers valuable insights into their dynamics and interactions, accurately predicting peptide stability remains challenging. This difficulty arises from the peptides’ inherent structural diversity and sensitivity to environmental conditions [91,135]. Machine-learning approaches have been applied to predict peptide stability versus gastrointestinal digestion using a training dataset of 109 peptides with known data from in vitro experimentally simulated gastrointestinal digestions [136].

### 2.5. Folding, Aggregation, and Peptide Conformation

The tendency of bioactive peptides to fold into specific conformations or aggregate can significantly impact their bioavailability and biological activity [17,137]. Aggregation can reduce the peptide’s solubility and decrease its ability to reach its target sites, potentially altering its functional properties [138,139,140]. Protein misfolding is associated with numerous disorders, including the neurodegenerative diseases Alzheimer’s and Parkinson’s [141,142,143]. By studying the principles of peptide stability, insights into the mechanisms underlying protein misfolding and aggregation can be gained. Furthermore, understanding the interplay between peptide folding and misfolding provides valuable information for designing strategies to modulate protein conformation and restore proper folding [144,145].

Conformation changes can expose or hide specific functional groups, altering the peptide’s ability to interact with other molecules [146]. Therefore, understanding the peptide’s three-dimensional structure is crucial for predicting and optimising its biological activity. Molecular modelling methods present advanced platforms for understanding folding mechanisms, aggregation tendencies, and conformational dynamics [147,148]. Notable molecular methods such as molecular dynamics (MD) simulations have been extensively employed to investigate peptide folding, revealing insights into the energy landscape that governs this process, with enhanced sampling MD methods allowing for a more comprehensive exploration of folding pathways compared to conventional MD simulations [149]. MD simulations can provide detailed Gibbs free energy landscapes that highlight the stability of native conformations and the kinetics of folding events [150,151].

Overall, even small structural modifications could influence peptide bioactivity [104]. Molecular modelling tools can provide insights into how structural characteristics influence bioactivity by considering the effects of any changes at a molecular level [99,152,153].

### 2.6. Peptide Structure Determination: Experimental Characterisation

Structure determination is crucial for thoroughly understanding the functions and bioactivity of peptides. Several experimental techniques have been at the centre of providing essential structural features of peptides, many of which rely primarily on spectroscopic techniques, notably, Nuclear Magnetic Resonance (NMR) spectroscopy [154,155,156]. NMR enables the direct study of peptide and small protein structures in solution or solid states, eliminating the need for crystallization. This provides a significant advantage over X-ray crystallography methods, particularly for peptides and small proteins that are typically difficult to crystallise. The ability of NMR to predict the structure of peptide structures under various environmental conditions also allows for the characterisation of the structural dynamics of peptides in different temperature, pH, and lipid composition conditions. Nonetheless, environmental variability can sometimes lead to NMR-derived peptide models diverging from their functional conformations. Other methods such as Circular Dichroism (CD) and Fourier Transform Infrared (FTIR) spectroscopy methods, often employed in combination, have also been employed to determine and characterise the secondary structure of peptides [157,158]. However, obvious issues with the small size and flexibility of peptides remain a challenge for the structural determination of peptides, often necessitating a multi-technique approach. In addition, experimental methods are also usually time-consuming and resource-intensive, making computational methods attractive.

## 3. Current Molecular Modelling Methods in Bioactive Peptide Discovery

**Figure 2:**
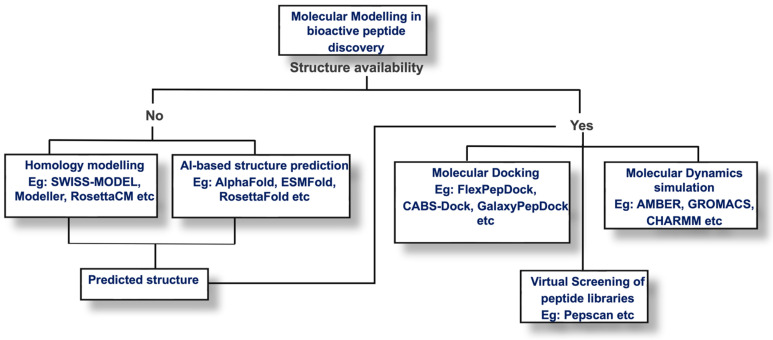
Molecular modelling methods for the discovery of bioactive peptides. The process begins with determining the availability of a peptide structure. If unavailable, structure prediction is performed using homology modelling or AI-based tools. For available structures, molecular docking and molecular dynamics simulations are employed. Virtual screening of peptide libraries can be integrated to identify potential candidates.

Integrating molecular modelling methods into peptide discovery pipelines has allowed for the prediction and analysis of the folding, stability, interactions, and binding affinities of peptides with target proteins [4,36,39,159,160,161]. Conventional molecular modelling methods that have augmented traditional peptide design and discovery processes over the years include homology modelling, molecular docking, virtual screening, and molecular dynamics simulations [4,36,160,162,163]. Figure 2 describes a simple decision criterion to select the appropriate molecular modelling method for the discovery of bioactive peptides.

### 3.1. Homology Modelling in Bioactive Peptide Discovery

Homology modelling is a technique that enables the prediction of the 3D structure of any given peptide, under the assumption that peptides with similar sequences will fold in similar ways [164,165]. It has become an indispensable tool in the discovery and characterisation of bioactive peptides, particularly when experimental structural data are lacking. The historically important methods are reviewed below to outline some of the important principles; however, the current most powerful methods use AlphaFold (see below, Section 4.1) with appropriate template homologues. Overall, the process of homology modelling involves three steps: template identification, sequence–template alignment, and model construction [165,166,167,168]. The accuracy of the predicted peptide structures from homology modelling, therefore, largely depends on the quality of the template employed, the similarity between the target and template proteins, and the refinement methods used [169]. It is generally preferred that a high sequence identity, with a minimum threshold of 30%, exists between the query structure and the template structure, as this is shown to translate to greater confidence in the homology [170,171]. Homology modelling is particularly useful when experimental methods, such as X-ray crystallography or nuclear magnetic resonance (NMR) spectroscopy, are either not feasible or too time-consuming [169,172,173].

Homology modelling has been employed in several efforts to discover bioactive peptides. For example, a recent discovery of a novel 37-residue antimicrobial peptide used a support vector machine (SVM) pipeline, with the peptide’s structure predicted by I-TASSER [174,175], a template-based method for protein structure and function prediction. The predicted structure was subsequently characterised for its anti-tumoural properties through further experimental evaluations [174]. Structural parameters from homology modelling in E. coli proteins and peptide binding data were also integrated to develop an algorithm that significantly outperformed either method alone in predicting peptide binding sites of the molecular chaperone DnaK [176]. The algorithm also performed well on a blind test set, effectively identifying binders and non-binders with no prior information. In another report, several trypsin-inhibitor peptides from the Cucurbitaceae family, characterised by conserved disulfide bonds and an arginine-rich active centre, were modelled using homology modelling based on the X-ray structure of MCTI-II from bitter gourd [177]. The peptides were analysed in both native and trypsin-bound states, with energy minimisation supporting their binding behaviour. The study concluded that evolutionary mutations in non-conserved regions modulate the inhibitory function of the peptides without altering their conserved structural fold or binding interactions. Homology modelling has also been previously employed to elucidate the structural properties that account for the antimicrobial activity of the protegrin family of antimicrobial peptides. Sixty-two (62) protegrin- and protegrin-analogue peptides were studied using homology modelling, and molecular descriptors were correlated with experimental activity against six (6) microbial species, as well as hemolytic and cytotoxic effects, revealing that structural characteristics, rather than sequence similarity alone, are key determinants of antimicrobial activity [178]. This provides valuable insights into optimising peptide design for enhanced therapeutic efficacy.

Despite its advantages, homology modelling faces challenges, such as the availability of suitable templates and difficulties in accurately modelling flexible regions like loops, as these often exhibit significant variability among homologous proteins [168,179]. In structures with greater flexibility, the established correlation between sequence and structural divergence is less clear due to structural changes occurring without corresponding sequence variations [180]. Also, the reliability of generated models is contingent upon rigorous validation techniques that assess their accuracy against experimental structures when available [181].

### 3.2. Molecular Docking

Molecular docking facilitates the prediction of how peptides interact with their biological targets [48,182]. Molecular docking simulates the binding of peptides to specific receptor sites, providing insights into the ability of a peptide to bind to receptors and into the specific potential interactions [160,183,184,185]. Molecular docking involves fitting a peptide or ligand into a binding site of a target protein, which may include the active site or other interaction regions, while predicting how well it binds, as indicated by an associated numerical score [40,186,187]. The process typically includes sampling multiple docking poses, which are then ranked based on their predicted binding energies [188,189,190]. Molecular docking simulations often incorporate the concept of multipose binding, thus recognising that certain interactions in certain protein–ligand complexes are characterised by multiple orientations or conformations rather than single, fixed poses [191]. Multipose binding also provides a more comprehensive and realistic representation of ligand–protein interactions, potentially leading to more accurate predictions and better-informed drug-design strategies [191].

Various strategies exist for peptide–protein docking, including template-based [192,193], local [194], and global docking methods, each with different levels of accuracy and computational cost, depending on the quality of input data [195]. Unlike docking algorithms for small molecules, which require minimal computational resources, docking algorithms for peptides are generally more computationally intensive, particularly in virtual screening experiments [38,49]. Moreover, docking programs do not provide a one-size-fits-all solution for peptide ligands. Traditional molecular docking programs such as AutoDock Vina and MOE-Dock [196,197,198] often struggle with ligands longer than three amino acids [38,199]. Therefore, using peptide-specific docking protocols is essential for achieving greater accuracy. These docking tools offer unique features to handle the complexities of peptide flexibility and protein surface interactions [200,201].

The ability of molecular docking to unravel structural mechanisms of peptide–protein interaction and thus guide the identification and design of novel potential bioactive peptides has been extensively explored. In very few instances, these reports are accompanied by experimental evaluations. For instance, two potential antioxidant peptides, Tyr-Gly-Arg-Asp-Glu-Ile-Ser-Val (YGRDEISV) and Leu-Asp-Leu-Val-Lys-Pro-Gln (LDLVKPQ), identified using liquid chromatography–tandem mass spectrometry, were shown through molecular docking to block the entrance to the active site of myeloperoxidase as a proposed mechanism of action [202]. Similarly, the binding mode of Phe-Asp-Gly-Asp-Phe (FDGDF) to superoxide dismutase (SOD) was unravelled via molecular docking to augment experiment efforts in characterising the binding mechanism. The docking analysis provided structural insights into the experimentally established antioxidant properties, highlighting crucial interactions and hotspot residues [203].

Molecular docking can also be followed by MD simulations to provide a more comprehensive understanding of peptide behaviour within biological systems. While molecular docking offers initial insights into binding interactions, molecular dynamics can elucidate the stability and dynamics of peptide–protein complexes over time [39]. The growing number of peptide–protein structures has enabled detailed characterisation, driving advances in peptide docking through diverse sampling techniques, which result in models that enhance predictions of binding specificity and aid inhibitor design [204].

Considering the vital role of peptide-mediated interactions in regulatory processes that involve dynamic protein interactions [205,206,207], modelling these interactions could unravel structural insights to guide the discovery and design of targeted inhibitors. The inhibitory mechanism of two peptides derived from camel and goat milk, IMEQQQTEDEQQDK and DQHQKAMKPWTQPK, identified using liquid chromatography–quadrupole time-of-flight mass spectrometry as potent inhibitors of pancreatic α-amylase (PAA), was characterized through molecular docking [208]. The analysis revealed that these peptides exhibit a strong affinity for the catalytic site of PAA, indicating their potential as effective inhibitors. In another study, five novel peptides were identified through LC-MS/MS and evaluated for their ACE-inhibitory activity [209]. The peptides, including Ala-Leu-Gly-Pro-Gln-Phe-Tyr and others, exhibited IC50 values ranging from 0.012 mM to 1.680 mM. Crucially, molecular docking studies played a key role in confirming the ACE-inhibitory effects, revealing that the peptides primarily interact with ACE through hydrogen bonding and electrostatic forces. Several molecular docking tools have been developed specially for peptide–protein docking, as highlighted in Table 1.

#### 3.2.1. Challenges of Peptide–Protein Docking

Unlike traditional protein–ligand or protein–protein docking, peptide–protein docking faces distinct difficulties primarily due to the inherent flexibility of peptides and the often unknown binding sites on target proteins [52,127,194,221,229,230]. This flexibility complicates the identification of favourable interactions, as conventional docking algorithms for small molecules often assume a fixed ligand conformation [210,231]. The challenge becomes even more complex when receptor flexibility is considered, as peptide–protein binding can induce conformational changes in the protein. These changes may involve side chain reorganisation and/or possible backbone rearrangement [194,232,233]. The lack of stable conformation of peptides prior to binding makes peptide–protein docking computationally expensive to fully sample the conformational space [127,229]. This limits the practical application of existing docking algorithms for high-throughput screening in drug discovery. The classical rigid docking approach of traditional molecular docking often fails to capture key structural adaptations, necessitating the incorporation of binding models such as induced fit and conformational selection in some docking approaches [234]. The concept of induced fit suggests that ligand binding triggers structural rearrangements in the protein, requiring flexible docking techniques to accommodate these changes [235,236]. Induced fit docking (IFD) can account for significant side-chain conformational changes and small backbone relaxation, and the approach has shown success, with some methods, like Schrödinger’s IFD-MD approach successfully predicting 85% of 258 protein–ligand pairs correctly [237]. On the other hand, the conformational selection model proposes that proteins exist in multiple pre-formed conformations, with ligands selectively binding to the most favourable state, highlighting the need for ensemble docking strategies [238]. Since many biological systems exhibit aspects of both models, integrating hybrid approaches that consider induced fit effects while leveraging conformational ensembles enhances the predictive power of docking algorithms and improves reliability in drug discovery applications [239,240,241,242,243].

In addressing the flexibility challenge, some docking tools, particularly global docking protocols, typically employ rigid-body docking by ignoring receptor flexibility, with an added advantage of low computational cost [194]. However, these tools would often allow side-chain flexibility in later modelling steps. Some advanced tools like Gold [244], which uses ensemble docking, and DynaDock [220], which uses soft potentials, attempt to mimic flexibility. Coarse-grained protein models, like CABS-Flex [245] and Rosetta FlexPepDock ab initio [226], can also be applied to capture large-scale backbone rearrangements, especially in disordered or loop regions near binding sites. For instance, Rosetta FlexPepDock ab initio generates coarse-grained representations of both the peptide and the receptor while simultaneously exploring rigid body orientations around the peptide-binding surface of the target.

Current scoring functions used in peptide–protein docking may also not adequately account for all relevant interactions between peptides and their targets. Many of these functions are optimised for small molecules or larger proteins, potentially leading to inaccurate predictions of peptide binding affinities [52,200,246]. Most peptide docking tools employ specific scoring functions to handle the complexities of peptides. For instance, Rosetta FlexPepDock uses the Rosetta energy function, MM-PBSA is used in HADDOCK and BiPPred [247], semi-empirical physicochemical scoring in pyDockWEB [248], and MD-derived binding energy in pepATTRACT. Other methods, such as CABS-dock, employ knowledge-based scoring functions. Structural clustering, the use of coevolutionary information, mutagenesis data, sequence-based predictions, and template-based comparisons have also been employed to improve model selection [193,249,250,251,252]. Hybrid approaches that combine mixed scoring functions with biological insights have been shown to yield the most accurate peptide–protein docking results [193,194,252].

Another challenge of peptide–protein docking is the instance of unknown or improperly defined peptide binding sites. This contrasts with protein–ligand docking, where the binding sites are usually characterised. Consequently, peptide–protein docking often requires a global search across the entire protein surface to identify potential binding modes, which increases computational complexity and time [210,253,254].

Peptide-mediated interactions also tend to be weaker and more transient than those involving larger ligands or proteins [255,256,257]. Many of these binding interactions are subtle regulators and appear evolutionarily optimised for low rather than high affinity [258]. The transient nature of these interactions also complicates experimental validation, as traditional methods like X-ray crystallography may not capture fleeting complexes effectively. The smaller interface between peptides and their protein partners results in lower binding affinities, making it difficult to accurately model these interactions using standard scoring functions [127,194,259].

#### 3.2.2. Clustering in Molecular Docking

Clustering enhances the efficiency and accuracy of molecular docking, particularly with flexible receptors/peptides and large datasets [260,261,262]. The clustering technique reduces computational complexity by condensing vast conformational ensembles, such as those from MD simulations, into a manageable set of representative structures. This allows for virtual screening against a diverse range of receptor/peptide states without docking to every single conformation. By grouping similar conformations and selecting representative structures, clustering helps capture essential receptor/peptide flexibility and improves docking efficiency, often leading to increased hit rates [250]. Peptide docking tools such as CABS-dock [263], HADDOCK [216], Cluspro PeptiDock [219], and FlexPepDock [226] incorporate clustering to enhance their accuracy. These cluster-based docking methods apply clustering algorithms to the ensemble of docked poses based on root mean square deviations of the structures [264]. Furthermore, clustering aids in the interpretation of results by grouping similar binding modes and identifying key conformational states, facilitating the analysis of ligand-receptor interactions. Overall, clustering is essential for streamlining computational demands, representing receptor flexibility, and improving the effectiveness of structure-based drug design approaches.

### 3.3. Virtual Screening of Peptide Libraries

Physical screening of peptide libraries is an important contributor to peptide development [265]. Virtual screening (VS) can search libraries of peptides, usually with docking software, to identify or up-rank the most promising peptides with high affinity for a protein target [43,266,267]. VS can also allow for the repurposing of peptides with known functions for new functions [268].

VS are generally divided into two main categories, namely structure-based virtual screening (SBVS) and ligand-based virtual screening (LBVS). SBVS utilises the three-dimensional structure of biological targets, obtained through X-ray crystallography, NMR spectroscopy, cryo-electron microscopy (cryo-EM), or computational modelling, to evaluate large libraries of peptide compounds [43,269,270,271]. SBVS allows for the prediction of binding affinities and poses of potential peptide ligands within the target’s binding site, effectively narrowing down candidates for experimental testing. SBVS can handle flexible binding sites and predict novel binding modes, which is particularly valuable for peptides with complex conformations [272,273]. On the other hand, LBVS methods, typically combined with docking and modelled on a known structure of a lead molecule complexed with a target protein, include similarity searching, pharmacophore modelling, machine-learning approaches, and shape-based screening. They leverage information from known active compounds to identify potential new bioactive peptides [274,275,276]. LBVS techniques are efficient in early-stage discovery, applicable to diverse targets (including those with unavailable experimentally defined 3D structures), and capable of handling flexible binding sites [277].

The efficiency and accuracy of virtual screening largely depend on how well the receptor’s binding pocket is characterised, with the binding pocket typically defined through molecular docking, based on the crystal structure of the proteins bound to ligands [278]. Although VS has become increasingly popular for identifying potential hits in small-molecule drug discovery, its application in the screening of peptide libraries has yet to gain momentum since the screening of peptides is relatively computationally intensive and requires 3D-structure of the protein and the appropriate scoring and search algorithms [267,268].

Several studies have successfully utilised VS to discover potential bioactive peptides, yielding some promising results. For example, one virtual screening approach using molecular docking screened over 1400 peptides, leading to the identification of macrocyclic peptide 22, which showed improved target affinity and biological activity [279]. These findings were validated through X-ray crystallography, further expanding the accessible chemical space for drug design. In another report by Amarasinghe et al., VS was employed to explore a vast library of nearly 380,000 non-natural amino acids, facilitating the identification of mutations at specific “sweet spots” on the peptide that could enhance its affinity for Keap1 [270]. Despite screening only a subset (less than 3%) of the full virtual library, the method successfully pinpointed mutations with the potential to improve protein–peptide binding. The study highlights the power of VS in rapidly evaluating large chemical spaces and postulates that scaling up the process could reveal even more effective peptide variants. In another report, a hierarchical structure-based virtual screening approach was employed to identify novel peptide inhibitors of human α-glucosidase [280]. Small di- and tripeptides, naturally present in the digestive system, were screened, with MD simulations used to evaluate their stability compared to the known inhibitor, acarbose. Four promising peptides with low RMSD variability were synthesised, and one (compound 2) demonstrated significant inhibitory activity in vitro. VS, combined with MD simulations, also played a crucial role in identifying and refining potential peptide scaffolds that closely resembled the three-dimensional shape and electrical properties of ATP-like compounds. These structures, upon further refinement and optimisation, could potentially serve as potent inhibitors of eIF4A1, thereby exhibiting promising antitumour effects. [281,282]. VS also played a pivotal role in identifying novel inhibitors of pancreatic lipase (PL), a key enzyme involved in triglyceride metabolism and linked to acute pancreatitis [283]. By screening a tripeptide library, the method successfully prioritised the top nine peptides for synthesis and in vitro testing, including two found to have strong inhibitory effects. Li et al. also identified 17 novel potential anti-inflammatory peptides from broccoli fermented by Lactobacillus strains through VS [284]. Augmented by in vitro assessments, it was further revealed that one of the peptides, SIWYGPDRP, showed the strongest inhibitory effect on NO release from inflammatory cells. Additionally, two other peptides, RFR and KASFAFAGL, effectively inhibited the secretion of TNF-α and IL-6, respectively. VS, combined with experimental validation, has also been applied in the discovery of a cyclic peptide inhibitor of thrombin from a library of 108,659 multiconformer cyclic peptides [285].

**Figure 3:**
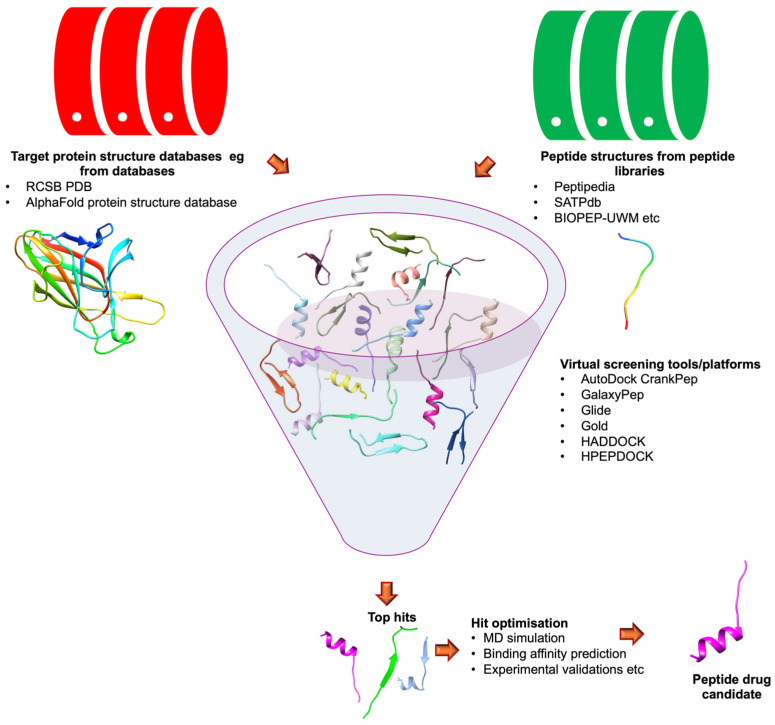
Schematic representation of the virtual screening-based discovery of bioactive peptides.

As represented in Figure 3, VS for bioactive peptides is a systematic approach that begins with the retrieval of peptides from peptide libraries, e.g, PepBank [286], PlantPepDB [287], Peptipedia [288], BIOPEP-UWM [289], Antimicrobial Peptide Database [290], StraPep [291], and CyclicPepedia [292]. Customised libraries can also be designed. This is followed by molecular docking simulations to evaluate binding affinities with target proteins. The peptides are then scored and ranked based on their predicted interactions. Additional filtering may be applied using extensive modelling techniques such as MD simulation and binding free energy calculations. Physicochemical properties may also be assessed to further refine candidate selection. Afterwards, the most promising candidates are synthesised and experimentally evaluated for biological activity. This streamlined process enhances the efficiency of identifying potential bioactive peptides while minimising resource expenditure.

Virtual screening thus provides a relevant computational approach to discovering bioactive peptides, enabling the rapid identification of potential candidates from vast peptide libraries while significantly reducing the time and resources required for experimental validation.

### 3.4. Molecular Dynamics Simulation

**Figure 4:**
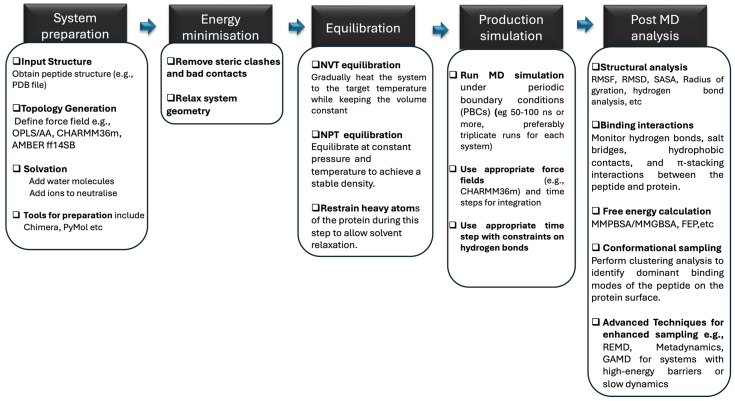
Overview of the MD simulation process for peptides.

Molecular dynamics (MD) simulation is a computational technique that can be used to study the physical movements of atoms and molecules over time to provide atomistic-level structural dynamics and thermodynamic properties of various systems, including biomolecules such as proteins and peptides [293,294,295,296]. MD simulation involves various steps, including initialisation, energy minimisation, equilibration, and production runs, as highlighted in Figure 4. This is followed by an analysis of structural stability, dynamics, and interactions [295].

In comparison to molecular docking, MD simulations provide a dynamic picture of protein–ligand interactions, offering several advantages over static molecular docking approaches. MD accounts for full atomistic flexibility, captures induced fit effects, includes explicit solvation, and provides insights into binding kinetics and conformational sampling [297,298,299]. In terms of scoring, MD enables a more reliable estimation of thermodynamic parameters, allows for ensemble averaging, incorporates entropic contributions, and can refine docking poses [185,294]. MD also captures time-dependent interactions and typically uses more advanced force fields [300,301]. While computationally more expensive, MD’s ability to provide a more realistic and comprehensive view of the binding process makes it an increasingly valuable tool in drug discovery, offering improved accuracy in predicting binding affinities and identifying potential drug candidates.

To perform MD simulations, several tools have been developed over the years, notably GROMACS [302], AMBER [303], CHARMM [304], NAMD [305], and OpenMM [306]. These tools, along with the use of graphic processing units (GPUs) on high-performance computing (HPC) systems, have significantly improved the speed and accuracy of simulations [307,308,309]. Some of these platforms allow for all-atom MD simulations, where every atom in a peptide and its environment are modelled, typically employing force fields such as AMBER or CHARMM and also enabling precise predictions of protein structures, binding modes of protein–ligand or protein–protein interactions [310,311].

An essential aspect of MD simulations is the accurate representation of the solvent environment. Solvent modelling plays a crucial role in the molecular modelling of peptides, influencing their folding, stability, and interactions [312,313,314,315]. Two primary approaches exist: explicit solvent models, such as TIP3P and TIP4P etc., which accurately capture hydrogen bonding and hydration effects but are computationally expensive [316,317], and implicit solvent models, like Generalized Born, Poisson–Boltzmann, and SASA, which offer faster calculations but lack explicit solvation details [318,319,320]. Key challenges include the trade-off between accuracy and computational efficiency, force field limitations, and the difficulty in capturing long-range electrostatic interactions [321]. Recent advances, such as hybrid solvation approaches, polarisable water models, and AI-driven techniques, aim to improve accuracy while maintaining computational feasibility [322,323,324,325]. Selecting an appropriate solvent model requires balancing computational cost with the level of detail needed for peptide simulations.

Recent advancements in computational power have enabled the MD simulation of large systems, reaching hundreds of thousands of atoms and millisecond-long time scales, thus enhancing the ability to study the complex conformational space of peptides and proteins [163,326,327]. In the study of peptides, MD simulation has also been very pivotal in several efforts towards the design and development of bioactive peptides. MD simulation has allowed for the investigation of conformational changes, flexibility, and interactions of peptides at an atomic level over time, aiding in the design of more potent and stable bioactive peptides [328,329,330]. This is particularly important in the context of peptide-based drug design, where small changes in sequence can lead to significant differences in biological activity [331]. For instance, in the study of antimicrobial peptides (AMPs), MD has elucidated the mechanisms of action of AMPs, revealing how they interact with bacterial membranes as well as the physicochemical properties that dictate their efficacy [332,333,334,335]. Simulations have also shown how AMPs can disrupt membrane integrity, leading to cell lysis, thus providing insights into the structural features that enhance their activity against resistant strains [336,337,338,339,340]. Using MD simulations, the stability and conformational rigidity of 12 stereo-engineered amphipathic peptides were tested against Gram-positive, Gram-negative, and antibiotic-resistant bacteria [341]. The results showed that syndiotactic peptides retained their designed electrostatic environment, exhibiting bactericidal activity and underscoring the potential of stereo-engineered peptides for therapeutic use [341]. MD simulations have also complemented experimental results to advance the Deno design of cell-penetrating peptides and further provide insights into mechanisms of CPP internalisation, membrane interactions, and peptide-induced membrane changes [342,343,344,345].

MD simulations have also furthered the atomistic-level understanding of protein–peptide interactions, enabling the exploration of their binding affinities and conformational changes upon interaction with target proteins [48,163], although they are often computationally expensive. To address this challenge of computational intensiveness, reports such as that of Chen et al. have proposed a new method that utilises high-temperature MD simulations with the RSFF2C force field to simultaneously predict peptide binding sites and poses [342]. By sampling thousands of binding events during microseconds of high-T MD, this approach accurately predicts peptide-receptor structures through density-based clustering analysis, achieving root-mean-square deviation (RMSD) values below 2.5 Å. This method was shown to also significantly improve the precision of protein–peptide docking, surpassing traditional approaches.

MD simulations have also been useful in modelling cyclic peptides, particularly in elucidating their solution structures, a crucial step toward quantitatively understanding the properties of cyclic peptides, using enhanced sampling techniques [346]. The reliability and accuracy of MD for simulating and understanding biomolecular systems, including peptides, are largely dependent on the conformational sampling methods (see Section 3.4.2 below) and the choice of force field (see Section 3.4.1 below) employed.

The integration of MD simulations and machine-learning (ML) methods has also sought to improve the accuracy of modelling peptides while aiding in the discovery of bioactive peptides [160,347,348,349]. Traditional MD methods, which typically rely on classical force fields and standard thermodynamic conditions, often struggle to capture complex, high-dimensional molecular interactions, but ML could offer flexible, unbiased solutions [350,351,352]. By learning from existing trajectories, ML accelerates convergence, guides simulations to relevant regions of configurational space, and improves exploration of rare events [348,353]. Additionally, ML enables the extraction of potential energy surfaces (PES) from quantum mechanical calculations, enhancing MD accuracy [354]. Its integration into Markov State Models (MSMs) further advances the analysis of MD trajectories, which can lead to a wider application in the study of the functional conformational changes of biological molecules [355].

MD simulation has, therefore, been useful in elucidating the dynamic behaviour and binding mechanisms of peptides, providing insights that can inform the design of peptide-based therapeutics and enhance our understanding of protein–protein interactions.

#### 3.4.1. Force Fields for MD Simulation Towards Bioactive Peptide Discovery

Selecting an appropriate MD force field is crucial for accurately modelling peptide behaviour, as it directly influences the reliability of simulations in capturing peptide dynamics, stability, and interactions with other biomolecules. Force fields determine the potential energy of a system based on the positions of its constituent atoms, using mathematical functions and parameters that describe various bonded and non-bonded interactions [301]. The accuracy and reliability of MD simulations are largely dependent on the choice of force field employed to accurately sample all the relevant conformations of the system [301]. Choosing the right force field is crucial in bioactive peptide discovery as it enables accurate prediction of peptide conformations and dynamics under physiological conditions, helps assess binding affinities between peptides and target proteins, and provides insights into peptide stability and folding mechanisms, key factors for their therapeutic effectiveness [301,356,357].

Although several force fields exist for MD simulations, most are not specifically tailored for peptides. Commonly used force fields include the AMBER force field [358,359], known for its detailed handling of proteins and nucleic acids; the CHARMM force field [360], which offers comprehensive coverage of biomolecules; GROMOS [361], designed for efficient simulations of biomolecules; and OPLS [362], primarily focused on small molecules but applicable to peptides. Recent advancements have introduced polarisable force fields like the Drude Polarisable Force Field and AMOEBA, which account for electronic polarisation effects that significantly influence molecular interactions [363,364,365]. Some of these force fields have been employed to study peptide systems; however, their efficiency in accurately parameterising peptides remains unclear. For instance, four different force fields—Amber ff99SB-disp [366], Amber ff99SB-ILDN [367], CHARM36IDPSFF [368], and CHARMM36m [369]—were comparatively investigated for their ability to capture the secondary structure element of five peptides previously suggested to possess polyproline II (PPII) structure [356]. Findings suggested that while all these force fields can generate conformational ensembles with polyproline II (PPII) structures, they differ significantly in their propensity to sample other secondary structures like β-sheets and random coils [356].

A few force fields have, however, been reported to consider peptides specifically. For instance, the ECEPP-05 force field parameterises both organic molecules and peptides where torsional parameters for peptides were derived by fitting molecular mechanics energies to quantum-mechanical energy maps of model peptides like Ac-Ala-NMe and Ac-Gly-NMe [370]. The reliability of ECEPP-05 was validated through simulations of small peptide crystal structures, which showed high accuracy with minimal deviations from experimental X-ray data, confirming its effectiveness in parameterising peptides [370]. The residue-specific force field (RSFF2) reported by Li et al. was shown to significantly improve agreement with experimental data in an MD simulation investigation of 256 peptides [371]. RSFF2 exhibited superiority over other derivatives of the Amber ff99SB force field in accurately modelling peptides by comparing their performance in reproducing experimental 3JHNHα scalar coupling constants for two-residue peptides. Although RSFF2 performed better, all force fields demonstrated similar limitations in capturing neighbouring residue effects (NREs), indicating potential challenges in their treatment of nonbonded interactions between adjacent side chains or terminal groups. RSFF2 and RSFF1 have also been shown to accurately identify the crystal-like conformations of cyclic peptides, with RSFF2 successfully identifying the most stable conformations for over half of the tested cyclic peptides [372]. In another study evaluating the ability of seven advanced force fields to reproduce experimental NMR data for 12 benchmark cyclic peptides, RSFF2+TIP3P, RSFF2C+TIP3P, and Amber14SB+TIP3P successfully captured the structure of 10 peptides, while Amber19SB+OPC demonstrated accuracy for eight peptides [373]. Regardless of these efforts, the challenge remains in parameterisation for non-standard amino acids and the computational demands of polarisable models [374]. The emergence of machine-learning force fields could address some of these challenges and consequently improve the accuracy of peptide parameterisation [375].

#### 3.4.2. Enhanced Sampling Methods

Enhanced sampling methods in molecular dynamics (MD) simulations address the limitations of conventional MD by overcoming energy barriers and exploring complex conformational landscapes [376,377]. Traditional MD often struggles with insufficient sampling due to rough energy landscapes with numerous local minima [378,379].

Notable enhanced sampling techniques like umbrella sampling [380], replica-exchange molecular dynamics (REMD) [381], metadynamics [382,383], accelerated molecular dynamics (aMD) [384], simulated annealing [385], and Gaussian accelerated molecular dynamics (GaMD) [386] amongst others, as shown in Table 2, are known to overcome the challenges associated with traditional MD [387]

Some of these enhanced methods have been employed to study peptide systems. For instance, Turner et al. employed REMD to investigate how amyloid-β (Aβ42) peptides interact with a platinum–phenanthroline complex (Pt(phen)) [388]. The REMD simulations revealed that the peptide adopts more compact conformations with increased helical content and reduced β-strand structure when bound to the platinum complex. REMD allowed for a thorough sampling of the diverse conformational states of the peptide, providing crucial insights into how metal complexes influence peptide structure and stability. In another study that introduced a method for calculating peptide dimer formation and dissociation rates, REMD simulations were employed [389]. By analysing continuous trajectories from replicas at various temperatures, the study accurately determined transition rates and validated the employed methods against alternative estimation techniques. The findings showed that REMD offers more than double the sampling efficiency compared to standard MD at lower temperatures. Clayton et al. also presented a hierarchical approach for exploring peptide conformational space by combining aMD and metadynamics [390]. Initially, aMD quickly scans accessible peptide states using an implicit solvent model, providing a qualitative view of conformations. Collective variables identified from the aMD were then used in well-tempered metadynamics with explicit solvent to accurately quantify free energy landscapes, despite the slower simulation speed. This combined method leveraged the strengths of both rapid exploration from aMD and precise free energy estimation from metadynamics, more efficiently and thoroughly characterising peptide states than conventional MD, which is often limited by slower timescales and insufficient sampling. Multiple simulated annealing–molecular dynamics (MSA-MD) has also been employed to improve peptide and miniprotein structure predictions [391]. MSA-MD was shown to significantly outperform MD and conventional simulated annealing–MD (SA-MD) in reaching native structures. The method successfully generated a cluster of near-native structures, demonstrating its enhanced effectiveness in structural prediction compared to traditional MD techniques. Wang et al. also incorporated GaMD to design a computational method called Pep-GaMD to overcome the limitations of conventional MD in simulating peptide binding and unbinding, which are hindered by long biological timescales and peptide flexibility [392]. Based on the GaMD technique, Pep-GaMD selectively boosts the peptide’s potential energy to better model its flexibility, while a dual-boost algorithm enhances the entire system’s potential energy. This approach enables the capture of repetitive binding and dissociation events, allowing accurate calculation of binding free energies and kinetics that align well with experimental data. Pep-GaMD significantly improves simulation efficiency and provides deeper insights into peptide–protein binding mechanisms compared to conventional MD. GaMD allowed for more efficient sampling of the conformational space to analyse large cyclic peptide libraries, to investigate how solvent affects the free energy landscape of cyclic lariat peptides, and showing that permeability is significantly influenced by N-methylation and solvent exposure [393].

**Table 2:** Table of enhanced sampling methods.

Method	Purpose and Application	Reference
Metadynamics	Accelerates the exploration of free energy landscapes by applying a history-dependent bias potential that discourages the system from revisiting previously explored states, pushing it out of energy wells to sample higher-energy states. This approach is useful in protein folding, ligand binding, and phase transition.	[394]
Umbrella Sampling	Enhances sampling in areas with high energy barriers by dividing the system into smaller, more manageable “windows” for easier sampling. Biasing forces are applied within each window, and the results are combined using the weighted histogram analysis method (WHAM) to reconstruct the free energy landscape. It is commonly applied in studying protein–ligand interactions, membrane fusion, and reaction mechanisms.	[380]
Replica Exchange Molecular Dynamics	Enhances sampling by running multiple simulations at different temperatures, periodically swapping configurations. Higher temperatures help the system overcome energy barriers, and the exchanges allow low-temperature replicas to benefit from broader sampling. This method is used in applications such as protein conformational sampling, studies of thermodynamic properties, and the exploration of phase transitions.	[395]
Adaptive Biasing Force (ABF)	Efficiently computes free energy profiles along a selected reaction coordinate by applying an adaptive biasing force to flatten energy barriers, promoting the exploration of rare conformational states. Over time, the force adjusts as the simulation progresses, making it useful for studying diffusion processes, chemical reactions, and molecular conformations.	[396]
Steered Molecular Dynamics (SMD)	Mimics experiments like atomic force microscopy (AFM) by applying an external force to specific parts of a system, driving it along a reaction pathway. It is used to study processes such as protein–ligand unbinding and the mechanical properties of proteins.	[397,398]
Accelerated Molecular Dynamics (aMD)	Lowers energy barriers by adding a boost potential to flatten energy wells, enabling faster sampling of conformations and helping the system escape local minima more easily. It is useful for studying large-scale conformational changes in proteins, molecular motors, and membrane simulations.	[399,400]
Path Sampling Methods	Transition Path Sampling (TPS) and Forward Flux Sampling (FFS) are methods designed to simulate rare events by focusing on sampling transition pathways directly, rather than the entire phase space. These techniques gather trajectories connecting initial and final states, particularly during rare transitions between stable states, and are applied in areas such as chemical reactions, protein folding, and nucleation processes.	[401]
Variationally Enhanced Sampling (VES)	Optimises biasing potential using a variational principle to enhance sampling in targeted regions. It constructs a free-energy landscape adaptively and applies an optimised bias potential for efficient exploration. This approach is particularly useful in applications such as protein folding, phase transitions, and complex chemical systems.	[402]
Gaussian Accelerated Molecular Dynamics (GaMD)	An extension of aMD that enhances conformational sampling while preserving the Gaussian distribution of the biased potential. It applies a boost potential similar to aMD but incorporates the constraint of maintaining Gaussian statistics, enabling accurate reweighting. This approach is useful for studying protein–ligand interactions and large biomolecular systems.	[386]

## 4. Machine Learning and Structural Modelling in Bioactive Peptide Discovery

Integrating artificial intelligence (AI) and structural modelling techniques can significantly accelerate the discovery of bioactive peptides. AI methods have enhanced the efficiency of predicting, designing, and validating peptides with specific biological functions using tools developed to specifically support bioactive peptide discovery [17,57,403].

AI approaches are able to utilise available datasets of known peptides and their biological activities to train models that can predict the structure, function, and efficacy of new peptide sequences and consequently aid in the optimisation of potential peptide-based therapeutics [404,405]. While these datasets provide valuable insights, their size is relatively modest compared to the extensive datasets typically used in AI applications, which may limit the scalability and robustness of predictions. Despite this, such approaches are instrumental in optimising potential peptide-based therapeutics. Using either simple machine-learning models, deep neural networks, or, more recently, protein large language models (pLLM), AI methods offer valuable predictions and hypothesis-generation capabilities that can significantly advance structural modelling efforts for bioactive peptide discovery [405,406,407,408,409,410].

Predicting and generating bioactive peptides computationally typically involves classifier methods, predictive systems, and deep-generative models like generative adversarial networks (GANs) and variational autoencoders (VAEs), and, more recently, diffusion-based models [411,412]. However, the successful application of these approaches requires the optimisation of processing workflows and the rigorous validation of predictive models to ensure accuracy and reliability [404,405].

The recent ability of AI methods to successfully predict the three-dimensional structures of proteins using primary sequences presents an opportunity for advancement in the structural modelling research into amino acid-based structures [404,413,414]. Several AI-based tools have been developed over the years to guide amino acid-based structure prediction, such as Raptor X [415], I-TASSER-MTD [416], trRosetta [417], and APPTEST [418], and more recently, RosettaFold [419], ESMFold [420], and AlphaFold [55]. Some of the recently developed AI-based tools (Table 3) have demonstrated reliable accuracy in predicting the structures of peptides, with many being AlphaFold-based implementations.

### 4.1. AlphaFold-Based Modelling and Bioactive Peptide Discovery

The introduction of AlphaFold, particularly its iterations AlphaFold 2 and the recently released AlphaFold 3, has marked a transformative phase in the modelling of protein structures and interactions, significantly impacting bioactive peptide discovery [429]. These deep learning-driven models have enhanced our ability to predict the three-dimensional structures of proteins and their complexes, facilitating the rational design of therapeutic peptides.

AlphaFold employs a two-step approach to predicting protein structures. It starts with a search of big sequence databases, including metagenomic sequences (e.g., Big Fantastic Database), to collect homologous sequences and build a Multiple Sequence Alignment (MSA) that provides evolutionary context and helps to identify conserved residues and potential structural features [55]. The core predictive power of AlphaFold lies in its advanced neural network architecture, which incorporates attention mechanisms that focus on relevant parts of the input data. This model predicts distances between pairs of amino acids and their relative orientations, ultimately generating a three-dimensional representation of the protein structure. ESMFold [420] is an alternative algorithm for modelling peptide structures. Instead of relying on MSA-based input, it uses hidden states derived from inference on a Protein Language Model (PLM). This PLM was pre-trained on a subset of UniRef-50 with a masked token prediction objective, enabling it to learn the conditional probability of a residue occupying a certain position given the context of the rest of the sequence. This method enables ESMFold to predict peptide structures with an accuracy slightly lower than AlphaFold2 but with a 60-fold speed-up. For peptide modelling with an interacting protein, a key benefit of AlphaFold2 over ESMFold is that it can model multiple interacting protein chains.

The application of AlphaFold in bioactive peptide discovery spans several areas [430,431]. One major application is predictive modelling, where AlphaFold accurately models peptide structures, allowing the prediction of how peptides fold and interact with target proteins [432]. This capability is particularly valuable for discovering therapeutic peptides with specific biological activities. For instance, AlphaFold was incorporated into a pipeline that accurately predicted peptide interactions with class I and class II MHC molecules, outperforming existing tools [432].

AlphaFold can accurately and rapidly model peptide–protein complexes without requiring multiple sequence alignment information for the peptide partner, as shown in an early implementation of AlphaFold monomer modelling (before the multimer version was available) by connecting the peptide to the receptor with a polyglycine linker [422]. AlphaFold demonstrated high-accuracy structure predictions for peptides ranging from 10 to 40 amino acids in length, particularly modelling α-helical, β-hairpin, and disulfide-rich peptides with accuracy comparable to or better than methods specifically designed for peptide structure prediction [433]. Similarly, Johansson-Åkhe et al. established that, aside from AlphaFold-Multimer’s ability to predict peptide–protein complexes with high quality exhibiting DockQ scores greater than 0.8, it could also determine whether a peptide and a protein could interact [434].

A number of AlphaFold implementations have been developed specifically for peptides. For example, HighFold [424] is an AlphaFold-derived model that incorporates specific structural features like head-to-tail circularity and disulfide bridges to accurately predict cyclic peptide structures. AfCycDesign is proposed to be able to predict cyclic peptide structures with an RMSD of less than 1.5 Å in many cases and to design a library of 10,000 macrocyclic 7-13mer peptides with high-confidence predicted structures [425]. They adapted the AlphaFold code to define an “offset” as the relationship of termini of the supplied linear peptide to be one residue apart, and this approach was further explored to design cyclic peptides for the MDM2/p53 complex and 12 other protein–peptide complexes [435]. Additionally, the integration of AlphaFold with generative models for peptide design has enabled the creation of novel peptides through deep generative models, such as GANs and VAEs, which explore vast chemical spaces to identify peptides with optimal binding affinities and enhanced stability for targeted drug delivery [436,437]. A generative model, such as a variational autoencoder or transformer, could be trained on existing peptide sequences to create novel candidates with desired properties. These sequences are then input into AlphaFold, which predicts their 3D structures, providing crucial structural information. This process allows for iterative optimisation, where structural feedback refines the peptide sequences generated by the model. The approach could enable rapid screening and ranking of candidate peptides based on both sequence and predicted structural features, ultimately leading to experimental validation of the top candidates. However, while this integration offers a faster and more cost-effective method for exploring peptide diversity, challenges include the computational cost of AlphaFold predictions and the accuracy of its predictions for short peptides.

AlphaFold’s computational efficiency also supports high-throughput screening, allowing for virtual screening of peptide libraries against target proteins. By predicting the binding affinities of thousands of peptide candidates, the most promising candidates for experimental validation can be efficiently prioritised [438,439,440].

Efforts to rank peptides towards determining which peptide would bind to a given protein target amongst a set of peptides has also recently been explored by new AlphaFold-derived methods such as Automated Pairwise Peptide-Receptor Analysis for Screening Engineered proteins (APPRAISE), a method that bridges the gap between protein structure prediction and binding prioritisation [441]. By utilising AlphaFold-Multimer and ESMFold for structural modelling, APPRAISE predicts the target-binding propensity of engineered proteins. It effectively classifies various engineered proteins, including adeno-associated viral vectors and peptides targeting SARS-CoV-2 and other receptors. Similarly, Chang et al. introduced a novel computational protocol that also leveraged AlphaFold’s capabilities to accurately predict protein structures and their interactions with peptides [442]. The assay enabled the identification of the highest affinity binder among a set of candidate peptides by modelling receptor structures in the presence of two peptides. The protocol effectively captured the higher affinity peptide in a bound state while depicting the other peptide in an unbound form when their individual structures are well predicted. Testing on six protein receptors with known experimental binding affinities confirms the assay’s efficacy, particularly for medium to strong peptide binders that adopt stable secondary structures upon binding.

AlphaFold has some limitations. Models showed consistently worse performance compared to native PDB structures when compared to four docking programs and two consensus techniques in a benchmark of 22 structurally known targets with known small-molecule ligands [443]. While AlphaFold-3 [444] permits a number of post-translational modifications of amino acids that are common in the PDB training dataset, more diverse noncanonical amino acids and unconventional cyclisation present challenges [430]. Thus, physics-based approaches like docking and molecular dynamics (MD) simulations remain essential in peptide drug discovery, additionally offering insights into binding mechanisms and the thermodynamic and kinetic properties of complexes. AlphaFold structures with the lowest RMSD do not always correspond to those with the highest pLDDT scores, and there are limitations in predicting Φ/Ψ angles and disulfide bond patterns [433], a limitation often ameliorated by relaxing the predicted structures with molecular modelling methods. Considering these limitations, synergistic integration of AI, physics-based methods coupled with experimental validation has the potential to transform peptide-based drug discovery. Overall, AlphaFold’s multifaceted applications are revolutionising the field of peptide discovery and could accelerate the design and identification of peptide-based therapeutics.

### 4.2. Structure-Aware Machine-Learning Methods for Predicting Peptide Bioactivity

Apart from the direct modelling of protein–peptide interactions, new machine-learning advances can leverage structural information for directly predicting bioactivity. These models can then be used to predict different relevant properties for the computational design of bioactive peptides with a series of desired physico-chemical properties.

These new methods take as input a peptide or protein structure, either experimental or predicted. They use that information to predict a target property of the peptide, such as antimicrobial activity. The methods represent the structures as undirected graphs where the nodes are the residues (or, in some cases, the atoms), and the edges represent the contact between those residues. Usually, an edge is defined when the distance between two given residues is lower than a predefined threshold. Both the nodes and edges can have different properties, describing them from naive one-hot encodings to more complex PLM representations. A framework for this, Esm-AxP-GDL, [427] has three basic steps: (1) prediction of the folding conformation of the peptide using ESMFold, (2) the folded conformation is translated into a graph representation where each residue constitutes a node and is represented by an embedding vector extracted from ESM and where the edges are defined between nodes within a given distance, and (3) a GNN is then used to learn to predict whether a peptide is antimicrobial or not. The main drawback of these approaches is that they require fairly large datasets, with the Esm-AxP-GDL framework having been trained on ~25,000 predicted antimicrobial peptide structures, most of which were less than 100 amino acids. It is not clear whether such use of structural information will enhance peptide bioactivity prediction since the performance of the method proposed did not outperform sequence-based methods for smaller peptides [427].

## 5. Conclusions

The field of peptide computational modelling and virtual screening is rapidly changing in response to developments in AI (AlphaFold and Protein Language Models). Such models are clearly important in accelerating scientific research regarding molecular interactions involving peptides. Nevertheless, the prospect of computational methods contributing in a major way to the clinical development of new peptide therapeutics is unclear. To date, most clinically used peptides are either microbial noncanonical peptides of non-ribosomal origin or human peptides that have undergone extensive chemical modifications to improve their stability. Thus, the key to the design of clinically useful peptides may lie in not only identifying and modelling bioactivity but also predicting peptides that have a reasonable bioavailability in the human body. Therefore, an outstanding challenge in this field is to improve the structural modelling of peptides with diverse chemical modifications and cyclisations.

The integration of molecular modelling techniques in bioactive peptide characterisation offers significant potential for advancing peptide therapeutics. Combining computational approaches such as molecular docking, molecular dynamics simulations, and QM/MM hybrid methods can enhance predictive accuracy, while AI-driven techniques improve structure–activity relationship predictions. Hybrid in silico-experimental workflows can streamline peptide screening, reducing trial and error. Additionally, enhanced sampling and multi-scale modelling provide deeper insights into peptide conformations and interactions. Future research should focus on optimising these methods for scalability, precision, and adaptability to non-traditional peptide structures, ultimately accelerating the discovery of next-generation bioactive peptides.

While the computational predictions presented in this review have, in many cases, been followed up by experimental analyses that appear to validate the computational method, this is a very piecemeal approach to methodology validation and does not comprise a benchmark for the field. Ideally, blinded benchmarking datasets would be provided prior to their publication so that the research community can rigorously assess the efficiency of different prediction methods, in a similar manner to the CASP competitions used to assess protein structure prediction [445]. However, designing or selecting such a validation dataset to have sufficient generalisability to diverse applications may represent a substantial challenge. The ultimate validation is whether such bioactive peptide discoveries have led to useful reagents, either to advance our understanding of molecular signalling or to act as lead therapeutic compounds. In general, peptides face some hurdles in translating into the clinic. For example, there is a vast treasure trove of ribosomally synthesised antimicrobial peptides, but these have not translated directly into the clinic. A challenge is how to build on the insights gained from the mechanism of action of novel bioactive peptides (such as dynobactin A, [446]) to develop lead compounds that act by the same mechanism but are more drug-like [446,447,448].

## Figures and Tables

**Table 1:** List of some prominent peptide–protein docking tools.

Tool	Synopsis	Server
HPEPDOCK 2.0 [210]	-Web server for blind peptide–protein docking that uses a hierarchical algorithm to efficiently sample peptide conformations and perform global docking across the protein surface.-Generates peptide structures with MODPEP and has demonstrated competitive performance in benchmarks, achieving high success rates in both global and local docking.-No licence required.-Available only as a web server.	http://huanglab.phys.hust.edu.cn/hpepdock/ (accessed on 12 December 2024)
CABS-Dock [211]	-Emphasises conformational flexibility for peptides and proteins using coarse-grained modelling, making it ideal for flexible docking scenarios.-Is available as a user-friendly web server and stand-alone tool, enabling efficient exploration of binding sites and poses.-Standalone executable available.-Free for academic and non-profit use under the MIT licence.	https://biocomp.chem.uw.edu.pl/CABSdock (accessed on 30 November 2024)
AutoDock CrankPep (ADCP) v1.1 [212]	-Tailored for peptide docking, offering flexible sampling of peptide conformations and achieving strong performance in predicting peptide–protein interactions.-Its emphasis on flexibility makes ADCP a valuable tool for exploring diverse binding modes in peptide–protein complexes.-Available under the GNU LGPL v2.0 OpenSource licence.-ADCP is part of the ADFR suite, which can be downloaded as a standalone package.-Not available as a standalone executable.	https://ccsb.scripps.edu/adcp/ (accessed on 30 November 2024)
GalaxyPepDock [213]	-Specialises in predicting peptide–protein interactions by refining docking poses using molecular dynamics.-Excels when target protein structures are known, providing reliable binding affinity predictions.-Freely accessible and does not require licence for use.-The Galaxy source code, which GalaxyPepDock is part of, is licenced under the Academic Free Licence version 3.0.-Standalone executable available.	https://galaxy.seoklab.org/cgi-bin/submit.cgi?type=PEPDOCK (accessed on 30 November 2024)
Rosetta FlexPepDock [214]	-High-resolution local docking algorithm that refines peptide binding modes within predefined binding sites.-Ideal for accurate predictions when detailed structural information about the binding site is available.-Rosetta licence required.-Command line options available.	http://flexpepdock.furmanlab.cs.huji.ac.il/overview.php (accessed on 30 November 2024)
PepCrawler [215]	-Refines peptide–protein interactions using a Rapidly Exploring Random Tree (RRT) algorithm.-Predicts and optimises peptide binding conformation and estimates binding affinity.-Useful for designing peptide inhibitors by refining docking solutions and improving peptide–protein binding.	Not available
HADDOCK2.2 [216]	-Suitable for peptide–protein docking, especially when binding site or interaction data is available.-Integrates biochemical and biophysical information to improve docking accuracy.-Standalone executable available.-Requires licence for non-commercial agreement.	https://www.bonvinlab.org/software/haddock2.2/ (accessed on 30 November 2024)
pepATTRACT [217]	-Flexible protein–peptide docking algorithm that performs efficient, coarse-grained global searches on the protein surface.-Enables rapid identification of potential binding modes for peptide–protein interactions.-Freely available.	https://bioserv.rpbs.univ-paris-diderot.fr/services/pepATTRACT/ (accessed on 30 November 2024)
PIPER-FlexPepDock [218]	-Fragment-based high-resolution peptide–protein docking protocol, integrating Rosetta’s fragment picker, PIPER for rigid-body docking, and FlexPepDock for flexible refinement.-Approach achieves accurate global peptide–protein docking, validated against X-ray crystallography data, enabling high-resolution modelling of peptide–protein interactions.-Requires a Rosetta licence for the FlexPepDock component.-PIPER component requires a separate commercial licence.	http://piperfpd.furmanlab.cs.huji.ac.il/ (accessed on 30 November 2024)
ClusPro PeptiDock 2.0 [219]	-An efficient method for docking peptide motifs to free receptor structures by conducting a motif-based search to retrieve structural fragments from the Protein Data Bank (PDB) that closely resemble the peptide’s bound conformation.-Utilises a Fast Fourier Transform (FFT)-based docking approach to perform global rigid-body docking of fragments to the receptor.-Freely available.-No standalone executable.	https://cluspro.org/peptide/index.php (accessed on 30 November 2024)
DynaDock [220]	-For docking peptides into flexible receptors, utilising the following two-step procedure: scanning the protein–peptide conformational space to identify approximate ligand poses, followed by refinement using optimised potential molecular dynamics (OPMD).-OPMD method employs soft-core potentials for protein–peptide interactions and a novel optimisation scheme, demonstrating significant improvements in sampling capability compared to conventional molecular dynamics and soft-core scaling methods.	Not available
AnchorDock [221]	-Automatically targets the docking search to the most relevant parts of the conformational space by precomputing the free peptide’s structure and by computationally identifying anchoring spots on the protein surface.-Free peptide conformation undergoes anchor-driven simulated annealing molecular dynamics simulations around the predicted anchoring spots.	Not available
MDockPep [222]	-User-friendly server for global docking of flexible peptides to protein receptors, starting from a peptide sequence and protein structure.-Docking results are scored using ITScorePeP, a statistical potential-based function, and validated with the peptiDB benchmark, showing high accuracy in both bound and unbound cases.-No licence required.-No standalone executable.	https://zougrouptoolkit.missouri.edu/mdockpep/ (accessed on 30 November 2024)
PEPFOLD3 [223]	-Predicting 3D structures of linear peptides (5–50 amino acids) in aqueous solution and peptide–protein interactions.-Supports both de novo and biased predictions, generating native-like peptide conformations when the interaction site is known.-No licence required.-No standalone executable.	http://bioserv.rpbs.univ-paris-diderot.fr/services/PEP-FOLD3 (accessed on 30 November 2024)
DINC 2.0 [224]	-Meta-docking strategy that overcomes this by incrementally docking ligand fragments, enabling accurate prediction of peptide-based inhibitors.-Improves upon the original by allowing docking of larger ligands (over 25 flexible bonds). -No licence required.-No standalone executable.	https://dinc-ensemble.kavrakilab.rice.edu/ (accessed on 30 November 2024)
GOLD [225]	-Automated program that predicts small-molecule binding to macromolecules by using a genetic algorithm.-Accounts for ligand flexibility and partial protein flexibility while ensuring displacement of loosely bound water molecules during binding.-Licence required.-No standalone executable.	Not available
Rosetta FlexPepDock ab initio [226]	-Allows for simultaneous docking and de novo folding of flexible peptides starting from an approximate peptide binding site specification.-Utilises the Rosetta fragments library and a coarse-grained structural model to effectively sample peptide conformations and rigid-body orientations on the receptor surface, followed by all-atom refinement to accurately model side-chain interactions.-Rosetta licence required.-Command line options available.-No standalone executable.	Not available
PaFlexPepDock [227]	-Parallel docking approach combining ab initio peptide folding, peptide docking, and flexible receptor refinement.-Showed improved interface modelling and energy funnel construction by refining receptor flexibility during docking.-Rosetta licence required.	Not available
PepComposer [228]	-Requires only the target protein structure and a rough definition of the binding site to start the design process.-Identifies peptide scaffolds with similar backbone arrangements and optimises sequences to best fit the target binding site.-Freely available.	Not available

**Table 3:** Common AI-based methods that use sequence information to predict 3D structures of peptides.

Tool	Synopsis	Availability
AlphaFold [55,421,422]	-Alignment-based deep learning model that predicts protein structures with high accuracy, including peptide conformations.-Adapted for cyclic peptides and can predict structures based on single sequences.	-AlphaFold Server is freely available for non-commercial research.-The code for AlphaFold3 is now downloadable for academic use.-Training weights on request for scientists with academic affiliations.
ColabFoldv1.5.5 [423]	-User-friendly interface implementing AlphaFold technology.-Allows batch predictions of structures in a single session.	-Web server-like interface implemented as a Google Colab notebook.-Can be installed and run locally.-Code is open-source and shared on GitHub at https://github.com/sokrypton/ColabFold (accessed on 30 November 2024).-Uses AlphaFold2 models, which typically include shared model weights for local deployment.-Can be run on high-performance computing (HPC) clusters.
Highfold v1.0 [424]	-AlphaFold implementation specifically designed for predicting cyclic peptide structures and their complexes.-Incorporates constraints like head-to-tail connections and disulfide bridges.	-The code for HighFold is shared on GitHub at https://github.com/hongliangduan/HighFold (accessed on 30 November 2024).-Uses AlphaFold2 models, which typically include shared model weights for local deployment.-https://github.com/sokrypton/ColabDesign/blob/main/af/examples/af_cyc_design.ipynb (accessed on).
AfCycDesign [425]	-AlphaFold implementation tailored for the rapid and accurate prediction and design of cyclic peptide monomers.-Addresses limitations related to insufficient training data.	-Available as an online web server through Neurosnap.-Implemented within the ColabDesign framework, which utilises AlphaFold for structure prediction and design.-Accessible via Google Colab, allowing users to run it without local installation.-Source code available at https://github.com/sokrypton/ColabDesign/blob/main/af/examples/af_cyc_design.ipynb (accessed on 30 November 2024).
RoseTTAFold [419]	-Deep-learning approach that excels in protein structure prediction and can be applied to peptide structures.-Provides high accuracy compared to traditional methods.	-Accessible via the Robetta web server.-Source code for RoseTTAFold is available on https://github.com/RosettaCommons/RoseTTAFold (accessed on 30 November 2024).-Model weights available and shared along with the code.
OmegaFold [426]	-Alignment-based deep learning model alternative to AlphaFold.	-Available as a standalone package.-Source code available on GitHub (OmegaFold GitHub Repository).-Model weights available and downloadable during execution.
ESMFold-2 [420,427]	-Deep learning built on Meta AI’s ESM-2 PLM.-Predicts 3D structures directly from amino acid sequences without relying on extensive homology (i.e., no alignments used in training or prediction).	-Available as a standalone package.-Open-source and does not require a commercial licence.-Code and pre-trained weights available on GitHub.-API available through BioLM.ai.
PepFlow [428]	-Deep-learning model trained on peptides of 15 or fewer residues to predict a wide range of dynamic folding patterns in peptides based on their energy landscapes.-Accurately predicts peptide structures and effectively recapitulates experimental peptide ensembles at a fraction of the running time of traditional approaches.	-Available as a GitLab repository at https://gitlab.com/oabdin/pepflow with code and documentation (accessed on 30 November 2024).

## Data Availability

Not applicable.
